# Preliminary Research on the Nonlinear Ultrasonic Detection of the Porosity of Porous Material Based on Dynamic Wavelet Fingerprint Technology

**DOI:** 10.3390/s19153328

**Published:** 2019-07-29

**Authors:** Xianghong Wang, Chenglong He, Wei Xie, Hongwei Hu

**Affiliations:** 1 Hunan Provincial Key Laboratory of Intelligent Manufacturing Technology for High-Performance Mechanical Equipment, Changsha University of Science & Technology, Changsha 410004, China; 2 Hunan Province Research Center for Safety Control Technology and Equipment of Bridge Engineering, Changsha University of Science & Technology, Changsha 410004, China

**Keywords:** dynamic wavelet fingerprint technology, porosity, nonlinear ultrasonic, second harmonic, nonlinear characteristic parameter

## Abstract

Porosity is an important characteristic of porous material, which affects mechanical and material properties. In order to solve the problem that the large distribution range of pore size of porous materials leads to the large detection errors of porosity, the non-linear ultrasonic testing technique is applied. A graphite composite was used as the experimental object in the study. As the accuracy of porosity is directly related with feature extraction, the dynamic wavelet fingerprint (DWFP) technology was utilized to extract the feature parameter of the ultrasonic signals. The effects of the wavelet function, scale factor, and white slice ratio on the extraction of the nonlinear feature are discussed. The SEM photos were conducted using gray value to identify the aperture. The relationship between pore diameter and detection accuracy was studied. Its results show that the DWFP technology could identify the second harmonic component well, and the extracted nonlinear feature could be used for the quantitative trait of porosity. The larger the proportion of the small diameter holes and the smaller the aperture distribution range was, the smaller the error was. This preliminary research aimed to improve the nondestructive testing accuracy of porosity and it is beneficial to the application of porous material in the manufacturing field.

## 1. Introduction

Porous material has the advantages of excellent mechanical properties such as being lightweight and having high specific strength, sound insulation, and good toughness. They are widely used in the construction [1], aviation [2], and other industries [3]. Porosity is closely related to porous material performance. The higher the porosity, the stronger the water absorption and sound insulation performance. Therefore, it is of great significance to detect the porosity. Porosity detection methods are mainly divided into destructive testing and nondestructive testing. The destructive methods for porosity include density measurement, water absorption, microscopy, and so on [4]. Since the process of destructive detection is complex, inefficient, and destructive to the specimen, nondestructive testing methods have been developed. At present, nondestructive testing methods for porosity mainly include a radial measurement, traditional linear ultrasonic measurement, and nonlinear ultrasonic measurement. The radial measurement uses the difference in intensity attenuation of X-rays when penetrating various parts of the porous material to achieve porosity detection [5,6]. However, only a pore diameter greater than 0.1 mm may be inspected using the radial measurement. Therefore, it is not as sensitive as the ultrasonic measurement and it endangers human safety as well because of radiation. The traditional linear ultrasonic measurement is based on the scattering characteristics of acoustic waves in the medium, which causes the attenuation of acoustic energy and the distortion of the frequency. The attenuation coefficient or the frequency domain characteristics can be used to evaluate the porosity [7]. The traditional linear ultrasonic measurement can detect large holes in material but is not sensitive to tiny holes [8]. However, the nonlinear ultrasonic measurement makes use of the nonlinear effect of ultrasonic waves interacting with tiny pores and performs the nondestructive evaluation on the porosity of the material. Zhang et al. [9] investigated the effect of plastic deformation on the nonlinear ultrasonic response of the austenitic stainless steel and showed that the nonlinear ultrasonic parameter increased with increasing the plastic strain. Shui et al. [10] used an AZ31 magnesium-aluminum alloy as a sample to measure the fatigue damage of the bonding layer and demonstrated that the nonlinear parameter increased with the increase of the fatigue cycle. Bermes et al. [11] used a nonlinear Lamb wave to detect the nonlinearity of the material to realize early damage detection and life prediction. The nonlinear ultrasonic measurement essentially reflects the influence of tiny pores on material nonlinearity, and the characteristic parameter is not limited by the size of the pores.

The extraction of the nonlinear characteristic parameter has a great effect on the accuracy of porosity detection. Generally, statistical characteristic parameters, like amplitude, mean values, RMS values and variance values, and frequency domain features, such as characteristic frequency and center frequency, can be extracted from the time domain and frequency domain, respectively. However, the above characteristic parameters in the time domain or frequency domain cannot significantly reflect the characteristics of the non-stationary signal. Therefore, feature extraction in the time–frequency domain is investigated.

Signal processing methods such as support vector regression [12] and spectrum analysis [11] are widely used in engineering, but they cannot accurately evaluate product performance. The wavelet transform is a multi-resolution signal analysis method, which can effectively extract time-frequency information. The dynamic wavelet fingerprint (DWFP) technique was developed from the wavelet transform for the effective feature extraction of significant details from signals in both the time domain and frequency domain. It quantifies the time–frequency characteristics of the signal by generating two-dimensional images resembling human fingerprints. Several studies have proved that DWFP technology can effectively extract the characteristics of an ultrasonic signal. For example, Hou et al. [13] extracted the number of white pixels in the fingerprint image to estimate the arrival time of various Lamb wave modes. In addition, Bingham et al. [14] extracted the gray circular feature fingerprint to realize the Lamb wave mode recognition. These studies definitely show that the DWFP technology has a high sensitivity to the time and frequency of ultrasonic detection signals. However, the DWFP technology is based on equal scale intervals and makes the wavelet coefficients have various frequency resolutions in different frequency sections. This is not beneficial to the accurate identification of nonlinear components. In order to overcome this deficiency, Jiao et al. [15] proposed an improved DWFP technology based on equal frequency intervals, and successfully applied it for the detection of fatigue microcracks.

As a result, the improved DWFP technology was used to study the porosity of the graphite composite for this paper and the relationship between the number of white pixels and the porosity was established. In Section 2, the DWFP technology with equal frequency intervals is advanced for the extraction of the nonlinear characteristic parameter in signals. In Section 3, the application of the DWFP technology in nonlinear ultrasonic signals is presented, and the effect of wavelet functions, the scale, and the white slice ratio are investigated to determine appropriate parameters. The relationship between porosity and characteristic parameters and the relationship between pore diameter and detection accuracy are studied in Section 4. The conclusions are drawn in Section 5.

## 2. DWFP Technology for the Extraction of the Nonlinear Characteristic Parameter

**Figure 1:**
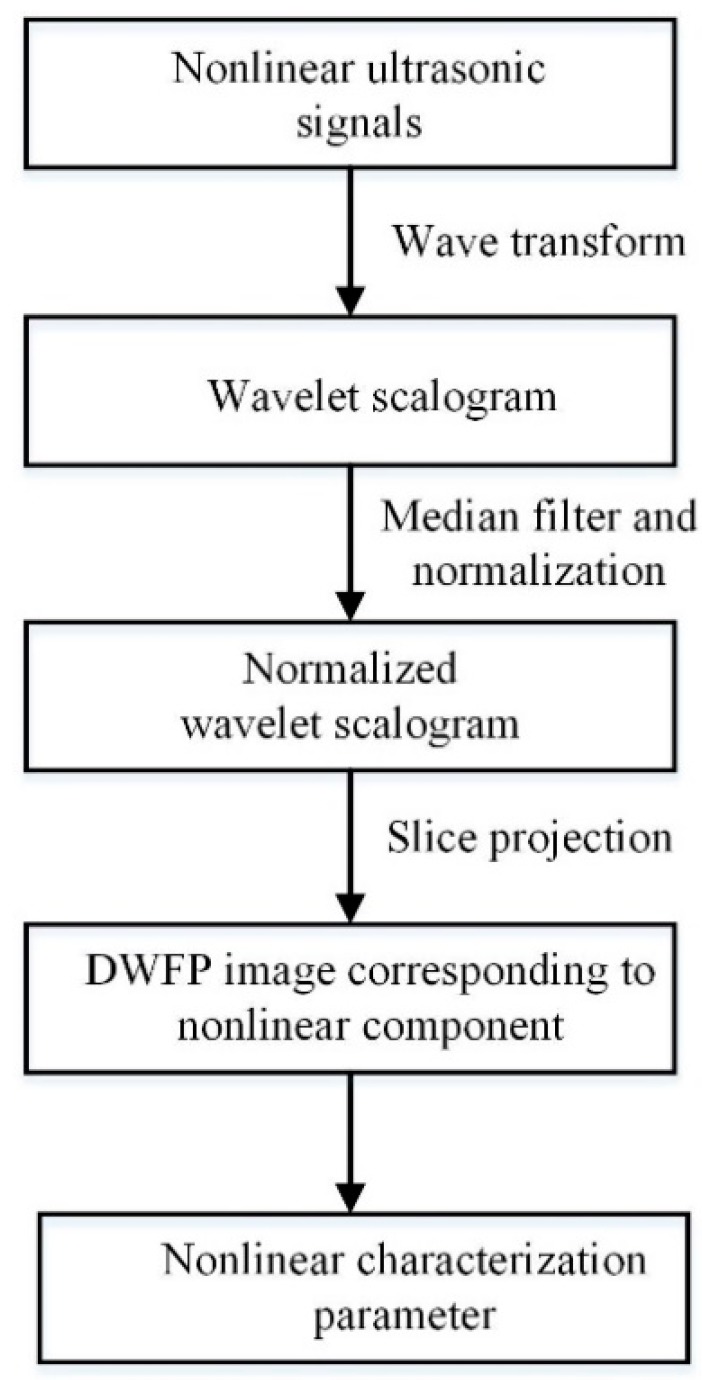
Flow diagram of extracting the nonlinear characteristic parameter using DWFP technology.

The DWFP technology is based on a wavelet transform to deal with the nonlinear ultrasonic signals and obtain the number of white pixels in the fingerprint image to extract the nonlinear effect. The calculation process is shown in Figure 1. First, the signal is subjected to an equal frequency interval wavelet transform to achieve a wavelet scalogram. The wavelet scalogram is the time–frequency characteristic image of signals, which can not only display the time–frequency features of signals, but also express some high-frequency components with relatively little energy. It is beneficial to the extraction of the nonlinear characteristic parameter [16,17,18]. Then, the wavelet scalogram is carried out in the form of median filtering to eliminate noise. Considering the influence of the fundamental frequency response on the nonlinear effect, the wavelet scalogram is normalized, and a slice projection operation is performed to obtain a fingerprint image within the second harmonic frequency range. Finally, the nonlinear characteristic parameter for porosity evaluation is extracted from the DWFP image.

### 2.1. Wavelet Transform with an Equal Frequency Interval

The continuous wavelet transform of the signal *x*(*t*) can be expressed as:

(1) $$WT_{x}(a,b)=\frac{1}{\sqrt{a}}\int_{-\infty }^{+\infty }x(t)\psi *(\frac{t-b}{a})dt$$

where *a* is the scale parameter and *a* > 0, *b* is the time parameter, and ψ(t) is the wavelet function. * corresponds to the conjugate. ψ*((t−b)/a) is formed via dilation and translation from the wavelet function [19,20]. The relationship between the scale *a* and the frequency *f* in the wavelet transform is:

(2) $$f=\frac{f_{a}}{a\times T_{}}$$

where *T* is the sampling period of the signal, and *f_a_* is the center frequency of the wavelet function. Therefore, the relationship between *a* and *f* in the wavelet transform is related to the wavelet function and the sampling period. If the wavelet transform is performed at equal intervals, the frequency of the wavelet transform will be unequal. In the low-frequency band, the scale is large and the frequency resolution is high; in the high-frequency band, the scale is small and the frequency resolution is low. In consequence, the low resolution in the high-frequency band affects the accuracy of feature extraction. In order to achieve the precise extraction of nonlinear high-frequency components, the DWFP technology is used at the following equal frequency intervals:

(3) $$\{f_{size}\}=\frac{\{1,2,3,\ldots ,size\}\times f_{p}}{size}$$

where *size* is the largest scale of the wavelet transform. *f_p_* is the highest frequency in the analysis, and its value should be greater than the second harmonic frequency. Therefore, the scale *a_size_* of the wavelet transform is:

(4) $$\{a_{size}\}=\frac{f_{a}}{\{f_{size}\}\times T_{}}$$

Using this scale series to perform wavelet transform on the signal, wavelet coefficients of equal-frequency resolutions can be obtained. Based on Equation (1), the wavelet scalogram of the signal is calculated as follows:

(5) $$SG_{x}(a,b)={\left|WT_{x}(a,b)\right|}^{2}$$

### 2.2. Definition of the Nonlinear Characteristic Parameter

To eliminate the noise caused by the experiment system and the environment, the obtained wavelet scalogram is subjected to a median filtering processing. Concerning the impact of the excitation signal on the nonlinear response, the wavelet scalogram is normalized as follows [21,22]:

(6) $$SG_{x}^{n}(a,b)=\frac{SG_{x}(a,b)}{S_{1}{}^{2}}$$

where $SG_{x}^{n}(a,b)$ is the normalized wavelet scalogram, and $S_{1}$ is equal to the maximum value of the wavelet scalogram in the fundamental frequency band of the excitation signal.

**Figure 2:**
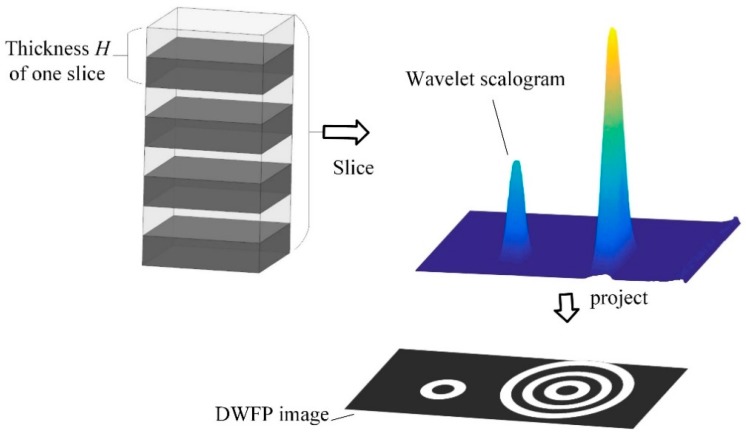
Slice projection schematic.

In the range of the second harmonic component, a periodic slice projection operation is executed on the normalized wavelet scalogram to generate a two-dimensional black and white fingerprint, as shown in Figure 2. Each slice has the same thickness of *H*, including two parts in black and white, where the ratio of the white portion to the thickness is *n*%. The wavelet scalogram cut by the white part is projected onto the two-dimensional time–frequency plane as white pixels (value is 1), and the wavelet scalogram cut by the black part is projected onto the two-dimensional time–frequency plane as black pixels (value is 0). In sequence, the slice projection operation of the scalogram in the entire amplitude range is completed, and then the three-dimensional distribution of the wavelet scalogram is converted into a two-dimensional image similar to the fingerprint.

It can be seen from Figure 2 that the number of fingerprints in the DWFP image is related to the amplitude of the wavelet scalogram. The larger the porosity, the larger the wavelet scalogram value, and the more fingerprints that are obtained. Therefore, the number of fingerprints can embody the layout of the wavelet scalogram amplitude and quantities in the white area can be used as the nonlinear characteristic parameter. The amount of white pixel points *S* in the DWFP image is defined as:

(7) $$S=\sum_{i=1}^{M}\sum_{j=1}^{N}L_{ij}$$

where *L_ij_* is the pixel value of row *i* and column *j* in the DWFP image; and *M*, *N* are the total number of pixels in the width and length directions of the image, respectively. The parameter *S* in the DWFP image is used to evaluate the porosity of the porous material in this paper.

## 3. Factors Influencing DWFP

The effects of the wavelet function and scale on the DWFP image were investigated because they are sensitive to the number of fingerprints. At the same time, the best white slice ratio can make the DWFP image clear. Thus, the influence of the wavelet function, scale, and white slice ratio are discussed in the following sections.

### 3.1. Experiment Research

Four graphite composites specimens S1–S4 with different porosity were prepared and their size was 170 mm × 170 mm × 4 mm. The mass volume method was applied for the specimen porosity detection and the porosities of S1–S4 were 0%, 24.2%, 35.1%, and 41.9%, respectively.

**Figure 3:**
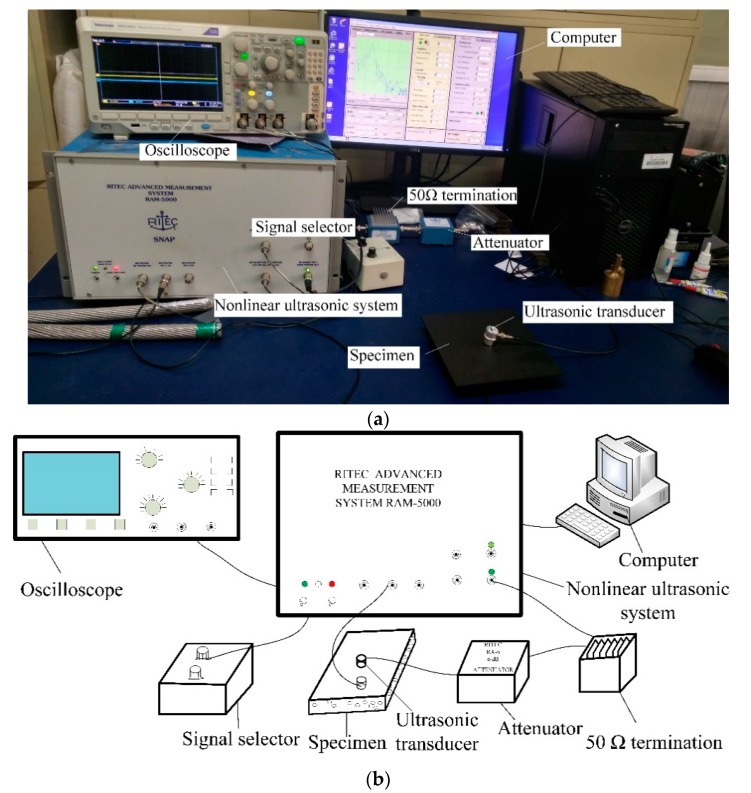
Experimental system: (**a**) physical map, and (**b**) schematic diagram.

The RITEC RAM-5000 SNAP nonlinear ultrasonic system (RITEC, Warwick, RI, USA) was employed in the experiment. The sinusoidal signal with 10 cycles at 2 MHz was generated using a transmitting transducer. The central frequencies of the transmitting and receiving transducers were 2.5 MHz and 5 MHz, respectively. The signal passed through a 50 Ω termination and an attenuator in order to suppress the transient behavior and high-frequency component from the amplifier. Then, the receiving signal entered the USB-UT350 (Ultratek, Creek, CA, USA) data acquisition card, and the fundamental wave and the second harmonic were extracted by the signal acquisition system. The physical map and schematic diagram of the nonlinear ultrasonic experimental system are shown in Figure 3. During the experiment, each sample was examined in five different areas, and the average of three test results per region was determined as the result for this region. The sampling frequency of the oscilloscope was 500 MS/s, the data length was 10,000, and the excitation voltage was 288 V.

### 3.2. Influence of Wavelet Function

**Figure 4:**
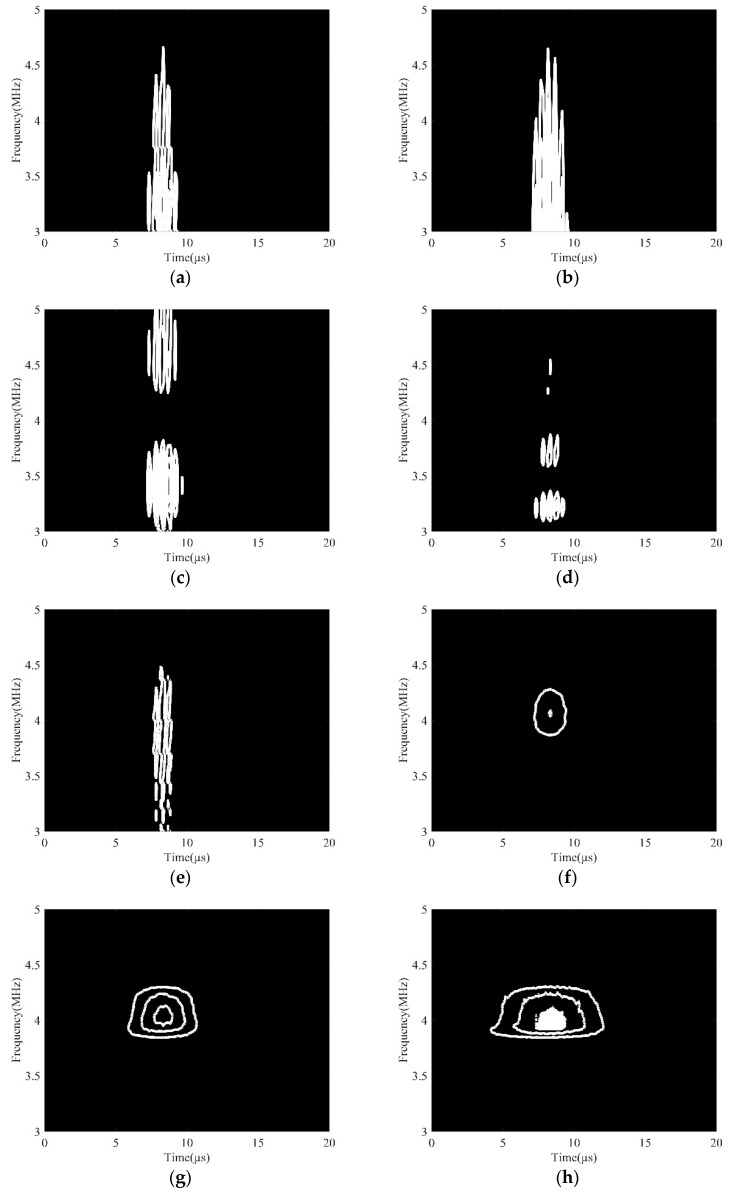
DWFP images of different wavelet functions. (**a**) Cgau4; (**b**) Haar; (**c**) Mexh; (**d**) Sym6; (**e**) Cmor1-1; (**f**) Cmor3-3; (**g**) Cmor5-5; (**h**) Cmor7-7.

In this section, the collected data with a porosity of 24.2% was used to study the influence of different wavelet functions on the DWFP image. Wavelet functions are families of functions satisfying prescribed conditions, such as continuity, zero mean amplitude, and finite or near finite duration [23]. Appropriate wavelet functions can make the results of the wavelet transform have good locality in the time domain and frequency domain. The scale was 1024. The DWFP images corresponding to the second harmonic component achieved by using various wavelet functions are shown in Figure 4. It clearly shows that the images had an apparent difference using different wavelet functions. When Haar, Cgau4, Sym6, Mexh, and Cmor1-1 wavelets were used, the DWFP images were very messy, and the time–frequency distribution information of the second harmonic component could not be accurately extracted. However, when choosing the Cmor3-3, Cmor5-5, and Cmor7-7 wavelets, the images have obvious fingerprint features and the center frequency of fingerprints was consistent with the second harmonic component frequency. Therefore, the Cmor3-3, Cmor5-5, and Cmor7-7 wavelets could be used for the extraction of the nonlinear parameter in ultrasonic signals. The reason for these three wavelet functions having obvious advantages is that these are single-frequency complex sinusoidal functions under a Gaussian envelope, which have a better local focus in both the time and frequency domains, and its waveforms are similar to the analyzed signal [24,25].

In addition, when the Cmor5-5 wavelet was selected, the amplitude of the wavelet scalogram was larger, and the number of fingerprints obtained was numerous, which made it easier to extract the nonlinear effect. Consequently, the Cmor5-5 wavelet was adopted in the study.

### 3.3. Influence of Scale Factor

**Figure 5:**
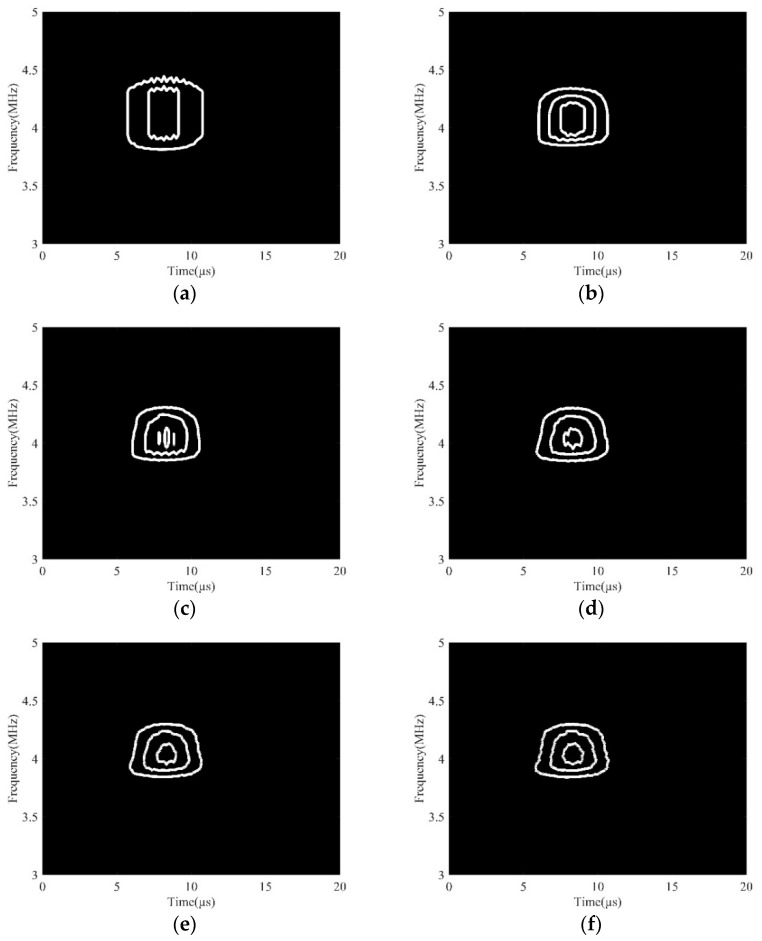
The DWFP images within different scales. (**a**) 64; (**b**) 128; (**c**) 256; (**d**) 512; (**e**) 1024; (**f**) 2048.

The DWFP images at different scales are shown in Figure 5. It shows that the image was unidentifiable at the small scale, and it was hard to obtain an accurate time–frequency distribution information for nonlinear components. When the scale was 1024, a clear DWFP image could be acquired because the DWFP technology performed wavelet transform with equal frequency intervals on the signal in a fixed frequency range [*f*_min_, *f*_max_]. The frequency interval of the wavelet scalogram is calculated as shown in the following equation:

(8) $$\Delta f=\frac{f_{\max}-f_{\min}}{size}$$

where [*f*_min_, *f*_max_] is the frequency distribution range of the wavelet transform, and *size* is the scale range. Equation (8) shows that when the frequency range is fixed, the frequency interval is inversely proportional to the scale range. As the scale increases, the frequency interval of the wavelet scalogram gradually decreases, and the frequency resolution is enhanced. In addition, the wavelet transform runtime will rise. Considering the relationship between frequency resolution and running time, the scale was determined to be 1024.

### 3.4. Influence of the White Slice Ratio

**Figure 6:**
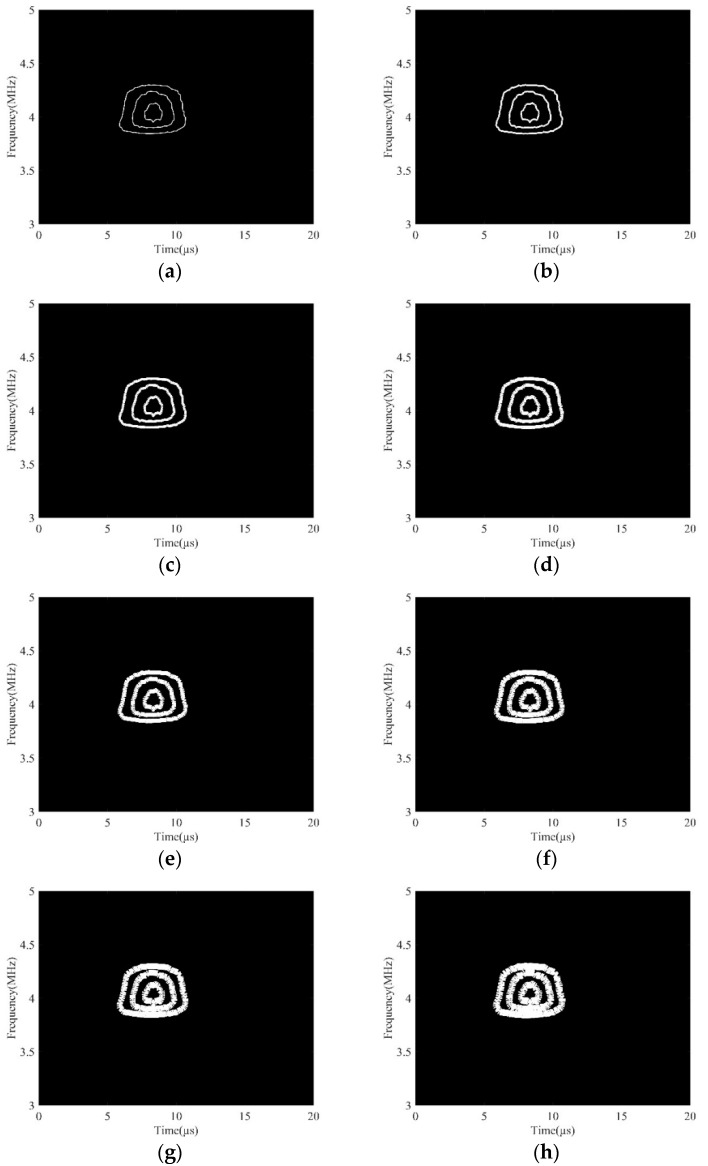
DWFP images of different white slice ratios. (**a**) 0.1; (**b**) 0.2; (**c**) 0.3; (**d**) 0.4; (**e**) 0.5; (**f**) 0.6; (**g**) 0.7; (**h**) 0.8.

The DWFP images at different white slice ratios are shown in Figure 6. It can be seen that when the proportion of white slice was low, the DWFP image was blurred; in contrast, when the proportion of white slice was high, the DWFP image became unrecognizable. When the proportion of white slices was 0.3, the image was clear. Therefore, the white slice ratio of 0.3 was selected as the optimal parameter for nonlinear feature extraction.

## 4. Results and Discussion

### 4.1. Analysis and Discussion

**Figure 7:**
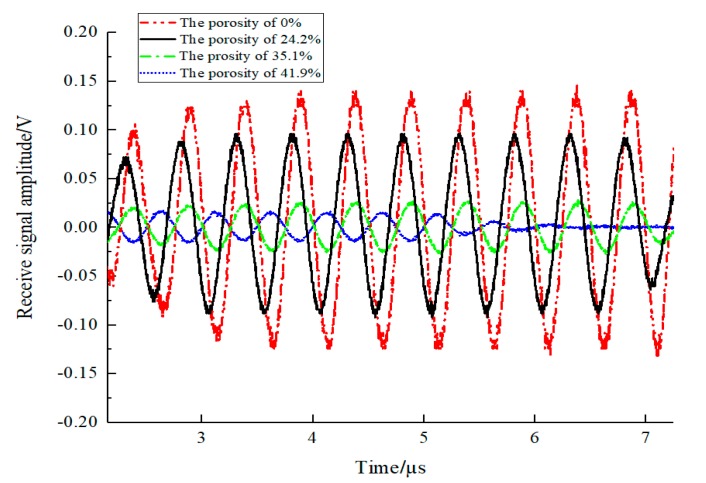
Time domain diagram of received signals for different samples.

The received signals for different samples are shown in Figure 7. It is clearly shown that the amplitude of the received signals increased with a decrease in the value of the porosity.

**Figure 8:**
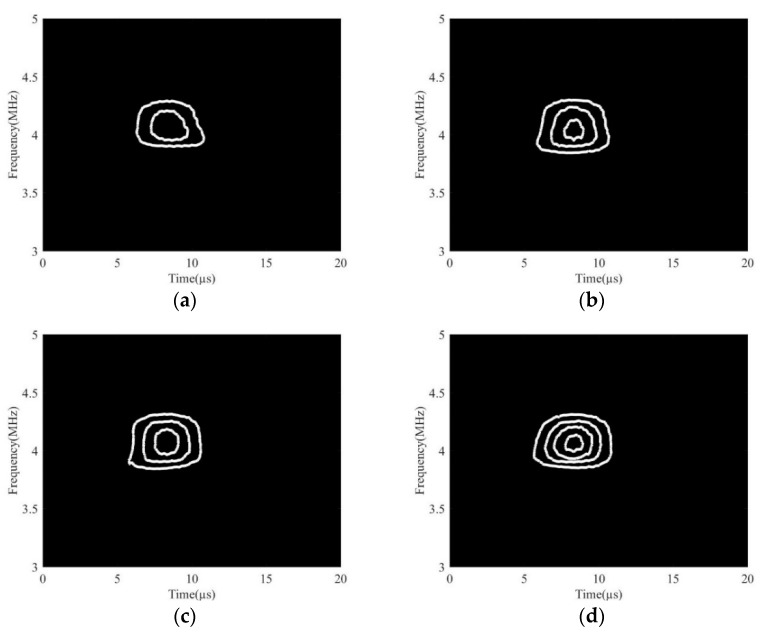
DWFP images corresponding to the second harmonic components for different porosities. (**a**) S1; (**b**) S2; (**c**) S3; (**d**) S4.

The second harmonic component was extracted because it was generated by the interaction between the ultrasonic waves and pores, which could indirectly reflect the porosity. The second harmonic results of the detection signals of four different specimens are shown in Figure 8. As the porosity increased, the number of fingerprints of the second harmonic component grew gradually, and the number of white pixels gradually increased.

In order to verify the accuracy of the DWFP method, the result was compared with that calculated by the attenuation method. The coefficient of the attenuation method was calculated according to:

(9) $$a=20\mathrm{lg}(V_{1}/V_{2})$$

where *V*_1_ is the amplitude of the fundamental wave and *V*_2_ is the second harmonic amplitude.

**Figure 9:**
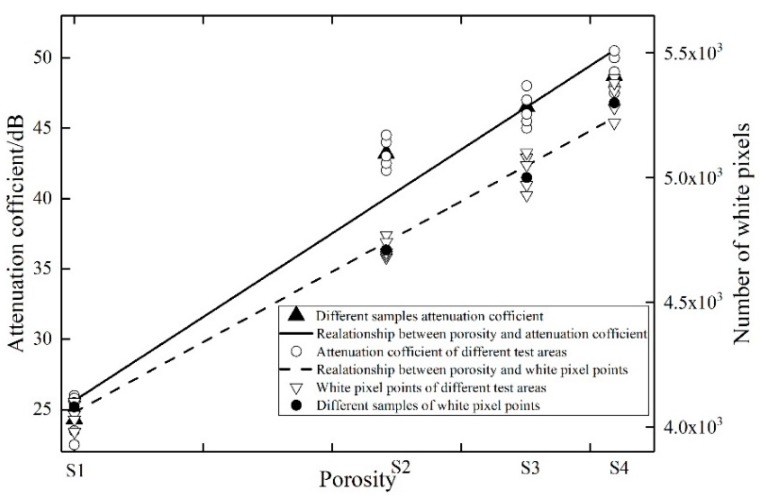
The relationships between the attenuation coefficient or the number of white pixels and the porosity.

**Figure 10:**
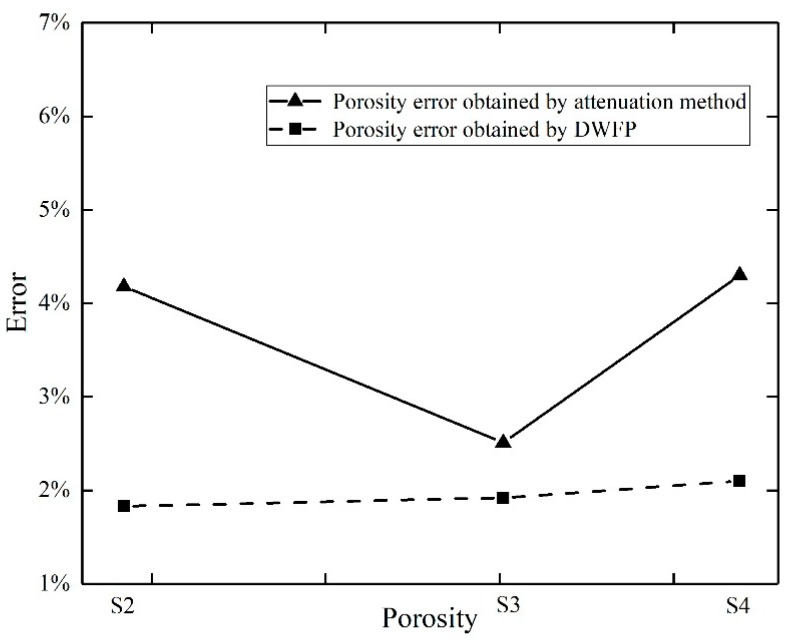
Error diagram obtained using two signal processing methods.

The relationships between the attenuation coefficient or the number of white pixels and the porosity are shown in Figure 9. It reveals that as the porosity increased, both the attenuation coefficient and the number of white pixels increased. In order to compare the accuracy of the two methods, the samples S2, S3, and S4 were used as verification specimens in turn. The testing errors of different methods are shown in Figure 10. It shows that the DWFP method had less error than the attenuation method.

### 4.2. Images Analysis

**Figure 11:**
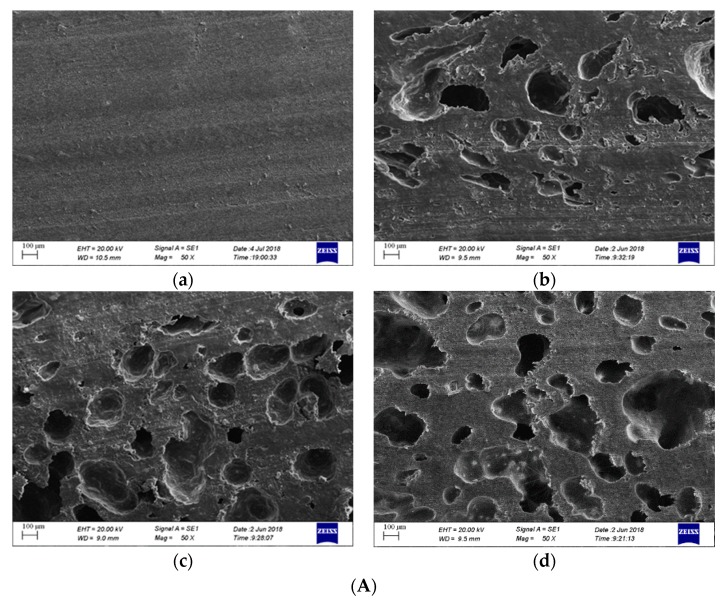

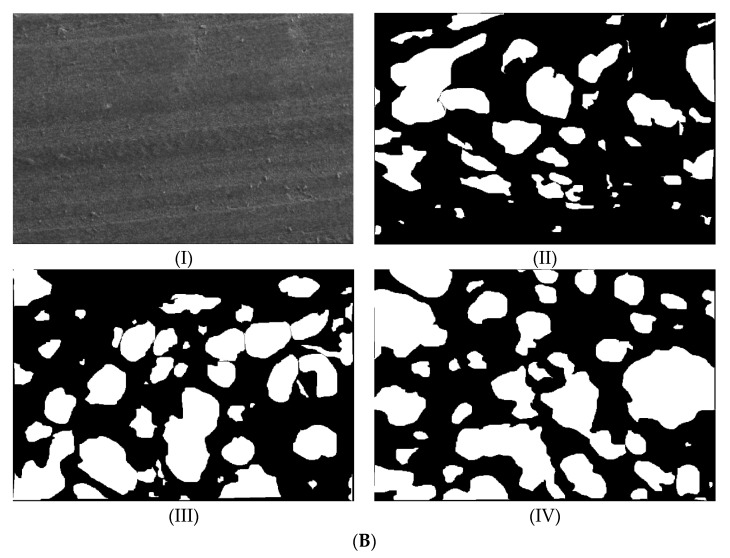
(**A**) SEM photos of different specimens, and (**B**) images processed using image processing. (**a**) S1; (**b**) S2; (**c**) S3; (**d**) S4; (**I**) S1; (**II**) S2; (**III**) S3; (**IV**) S4.

**Figure 12:**
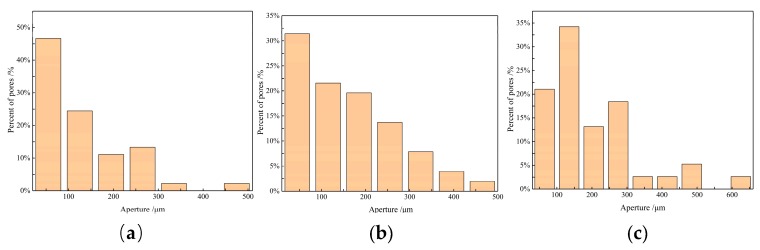
The histogram of the aperture distribution for the different samples. (**a**) S2; (**b**) S3; (**c**) S4.

The scanning electron microscope (SEM) photos of each sample are shown in Figure 11A. Then the SEM photos were conducted using gray value to identify the aperture. The parameters, such as average aperture and hole area, were counted. The processed images are presented in Figure 11B and the statistical results of the parameters are shown in Table 1. It can be seen from Table 1 that the average aperture of sample S3 was 132.85 µm and the standard deviation was 101.78, and the testing error was 1.83%. The standard deviation and average aperture of samples S3 and S4 were larger than those of S2, and their testing error was 1.92% and 2.1%, respectively. That is, with the increase of the standard deviation and average aperture, the testing error increased. The histogram of the aperture distribution for the different samples is shown in Figure 12. It shows that when the diameter of pores was less than 100 µm in the large proportion, the detection error was small. Therefore, the pore diameter had important effects on the porosity detection.

## 5. Conclusions

In this paper, graphite composites with different porosities were used as the specimen. In addition, the nonlinear ultrasonic signal analysis based on DWFP technology was investigated regarding porosity detection. The results indicate that:

(1) Wavelet function, scale, and white slice ratio had effects on the ultrasonic nonlinear extraction. Thus, the values of these three parameters must be chosen.
(2) The non-linear ultrasonic testing technology could exactly detect the porosity. The detection results of the nonlinear characterization method based on DWFP was more accurate than the conventional attenuation method in measuring porosity.
(3) The relationship between the statistical information of pores—such as average aperture, the standard deviation, and the histogram of the aperture distribution—and the errors of porosity were obtained. It was found that the pore diameter had an important influence on the detection results. The larger the proportion of small diameter holes was, the smaller the error was. Furthermore, the larger the aperture distribution range was, the larger the error was.

In the future, we will prepare a large number of samples to test and use different liquid media as coupling agents to measure, find the liquid medium with the smallest nonlinear residue, perform quantitative analysis, and eliminate it in the test results to reduce the influence of coupling agent. We will also establish a three-dimensional pore finite element simulation model to study the effect of pore morphology on nonlinear effects.

## Figures and Tables

**Table 1:** The statistical results of parameters.

Specimen Number	Testing Error of the DWFP Method	Testing Error of the Attenuation Method	The Minimum of the Aperture (µm)	The Maximum of the Aperture (µm)	Standard Deviation of the Aperture	Average Aperture (µm)
S2	1.83%	4.18%	20.85	507.29	101.78	132.85
S3	1.92%	2.51%	12.29	478.49	109.72	155.15
S4	2.1%	4.3%	31.11	635.18	134.5	197.35

## References

[1] Craig S., Grinham J. (2017). Breathing walls: The design of porous materials for heat exchange and decentralized ventilation. Energy Build..

[2] Kondrashov S.V., Gurevich Y.M., Popkov O.V. (2017). Broadband radio-absorbing materials based on porous composites with carbon nanotubes. Polym. Sci. Ser. D.

[3] Rashidi S., Esfahani J.A., Rashidi A. (2017). A review on the applications of porous materials in solar energy systems. Renew. Sustain. Energy Rev..

[4] Wits W.W., Carmignato S., Zanini F., Vaneker T.H. (2016). Porosity testing methods for the quality assessment of selective laser melted parts. CIRP Ann..

[5] Ziółkowski G., Chlebus E., Szymczyk P., Kurzac J. (2014). Application of X-ray CT method for discontinuity and porosity detection in 316L stainless steel parts produced with SLM technology. Arch. Civ. Mech. Eng..

[6] Maskery I., Aboulkhair N.T., Corfield M.R. (2016). Quantification and characterization of porosity in selectively melted Al-Si10-Mg using X-ray computed tomography. Mater. Charact..

[7] Yusof M.F.M., Kanaruzaman M.A., Lshak M. (2017). Porosity detection by analyzing arc sound signal acquired during the welding process of gas pipeline steel. Int. J. Adv. Manuf. Technol..

[8] Chen J., Ren J., Yin T. (2016). Nondestructive evaluation of notched cracks in mortars by nonlinear ultrasonic technique. Nondestruct. Test. Eval..

[9] Zhang J., Li S., Xuan F.-Z., Yang F. (2015). Effect of plastic deformation on nonlinear ultrasonic response of austenitic stainless steel. Mater. Sci. Eng. A.

[10] Shui G., Wang Y.-S., Huang P., Qu J. (2015). Nonlinear ultrasonic evaluation of the fatigue damage of adhesive joints. NDT E Int..

[11] Bermes C., Kim J.-Y., Qu J., Jacobs L.J. (2008). Nonlinear Lamb waves for the detection of material nonlinearity. Mech. Syst. Signal Process..

[12] Zuo Q., Zhu X., Liu Z. (2019). Prediction of the performance and emissions of a spark ignition engine fueled with butanol-gasoline blends based on support vector regression. Environ. Prog. Sustain. Energy.

[13] Hou J., Leonard K.R., Hinder M.K. (2004). Automatic multimode Lamb wave arrival time extraction for improved tomographic reconstruction. Inverse Probl..

[14] Bingham J., Hinders M., Friedman A. (2009). Lamb wave detection of limpet mines on ship hulls. Ultrasonics.

[15] Lv H., Jiao J., Meng X., He C., Wu B. (2017). Characterization of nonlinear ultrasonic effects using the dynamic wavelet fingerprint technique. J. Sound Vib..

[16] Hee L.M., Leong M.S., Lnayat-Hussian J.I. (2015). Diagnosis of blade fault based on wavelet scalogram and blade pass vibration signature analysis. J. Vib. Eng. Technol..

[17] Li H., Xu F., Liu H., Zhang X. (2015). Incipient fault information determination for rolling element bearing based on synchronous averaging reassigned wavelet scalogram. Measurement.

[18] Ren Z., Zhou S., Gong M., Li B., Wen B. (2015). Crack fault diagnosis of rotor systems using wavelet transforms. Comput. Electr. Eng..

[19] Mallat S. (1999). A Wavelet Tour of Signal Processing.

[20] Zala C.A., Barrodale I., McRae K.I. (1988). High resolution deconvolution of ultrasonic traces. Signal Processing and Pattern Recognition in Nondestructive Evaluation of Materials.

[21] Jhang K.-Y., Kim K.-C. (1999). Evaluation of material degradation using nonlinear acoustic effect. Ultrasonics.

[22] Liu M., Kim J.-Y., Jacobs L., Qu J. (2011). Experimental study of nonlinear Rayleigh wave propagation in shot-peened aluminum plates—Feasibility of measuring residual stress. NDT E Int..

[23] Chaudhary M., Dhamija A. (2013). A brief study of various wavelet families and compression techniques. J. Glob. Res. Comput. Sci..

[24] Cao Y., Liu M., Yang J. (2018). A method for extracting weak impact signal in NPP based on adaptive Morlet wavelet transform and kurtosis. Prog. Nucl. Energy.

[25] Amarnath M., Sujatha C. (2015). Surface Contact Fatigue Failure Assessment in Spur Gears Using Lubricant Film Thickness and Vibration Signal Analysis. Tribol. Trans..

